# Access to health care and employment status of people with disabilities in South India, the SIDE (South India Disability Evidence) study

**DOI:** 10.1186/1471-2458-14-1125

**Published:** 2014-11-01

**Authors:** Murthy Venkata S Gudlavalleti, Neena John, Komal Allagh, Jayanthi Sagar, Sureshkumar Kamalakannan, Srikrishna S Ramachandra, South India Disability Evidence Study Group

**Affiliations:** South Asia Centre for Disability Inclusive Development and Research, Indian Institute of Public Health, Public Health Foundation of India, ANV Arcade, 1 Amar Cooperative Society, Kavuri Hills, Madhapur, Hyderabad 500033 India

**Keywords:** Diabetes mellitus, Depression, Disabled persons, Health services accessibility, Health care disparities, India

## Abstract

**Background:**

Data shows that people with disability are more disadvantaged in accessing health, education and employment opportunities compared to people without a disability. There is a lack of credible documented evidence on health care access and barriers to access from India. The South India Disability Evidence (SIDE) Study was undertaken to understand the health needs of people with disabilities, and barriers to accessing health services.

**Methods:**

The study was conducted in one district each in two States (Andhra Pradesh and Karnataka) in 2012. Appropriate age and sex-matched people without a disability were recruited to compare with people with disability who were identified through a population-based survey and available government disability records by trained key informants. These people were then examined by a medical team to confirm the diagnosis. Investigators administered questionnaire schedules to people with and without a disability to harness information on employment and health service access, utilization and barriers.

**Results:**

A total of 839 people with disabilities and 1153 age and sex matched people without a disability, aged 18 years or more were included. People with disability had significantly lower employment rates. On univariate analysis, people with disability (18.4%) needed to visit a hospital significantly more often in the preceding year compared to people without a disability (8.8%) (*X*^2^- 40.0562; P < =0.001). However adjusted odds ratios did not show a statistically significant difference. Significant differences were also observed with respect to past hospitalization. People with disabilities had 4.6 times higher risk of suffering from diabetes and 5.8 times higher risk of suffering from depression compared to people without a disability and the risk was significantly higher in males compared to females with disability. People with disability faced significantly more barriers to accessing health services compared to people without a disability. Barriers included ignorance regarding availability of services, costs of services and transportation.

**Conclusions:**

This study highlights the challenges that people with disability face in accessing health-care and employment opportunities. The study findings have public health implications and should be used for planning need-based appropriate strategies to improve health care access for people with disabilities.

## Background

The World Health Report estimates that 15% of the global population has some degree of disability, defined in terms of functional limitation [1]. However analysis of data from 15 low and middle income countries shows that the prevalence varied significantly from 3.08% in Laos to 16.21% in Bangladesh [2]. The difference in prevalence could be due to differences in prevalence of the underlying conditions causing disability, differences in demographic characteristics like age and contextual factors and cultural interpretation of the meaning of disability [3].

Rates of disability are increasing due to population ageing and increase in chronic health conditions [1]. People with disability constitute one of the most marginalized and socially excluded groups in any society [3]. Studies also show a positive correlation between disability and poverty [3–5]. Data from the World Health Survey (2002–2004) from 54 countries reveals that disability prevalence is much higher in lower and lower-middle income countries compared to high income countries [6]. The exact magnitude of disability in India is not known as different definitions have been used by different groups. The World Bank estimated that 8-10% of the Indian population was living with disability in 2003 [7]. However the Census of India, 2011 reported the prevalence to be 2.21% [8].

The World Report on Disability highlights the fact that many people with disability do not have equal access to health care, education and employment [1]. The Report also emphasizes that people with disabilities have less access to health care services and therefore experience unmet health care needs [1]. Data shows that people with disabilities have greater unmet needs and services than their counterparts without disabilities [9]. Similar findings have been reported in a number of countries [10–12]. People with disabilities have difficulty in accessing quality health care [13]. This also results in people with disability seeking health care from unqualified practitioners in some countries [14].

Studies show that the commonest reason why people with disabilities do not access health care when it is needed is due to the cost of health care [1, 10, 15]. At the same time, studies also show that despite all their needs not being met, people with disabilities use hospital services significantly more compared to people without a disability [11, 16–19]. An extensive analysis shows that people with disabilities consistently had higher total health expenditures, out-of pocket (OOP) spending, and burden compared with their counterparts without disabilities [20]. The World Report on Disability also emphasizes that affordability of health services and transportation are two main reasons why people with disabilities do not access needed health care in low-income countries: 32-33% of non-disabled people are unable to afford health care compared to 51-53% of people with disabilities [1].

Evidence also shows that women with disability are most disadvantaged in most societies [2, 6, 11, 15, 21–27]. Over the past decade research addressing health of women with disabilities and addressing topics such as access to care, health care utilization, and the prevention of secondary conditions has been on the rise [28, 29]. However there is a distinct lack of evidence from population-based studies on the rates of access or unmet need for health services among people with disabilities in India. The present study was therefore undertaken to understand the health needs of people with disabilities, and barriers to accessing health services, as the provision of universal health care should address the needs of the most marginalized sections of the society if it has to be successful.

## Methods

The present study was conducted over a ten month period in 2012, to identify people with disability and ascertain their employment and health status, and access to health care and health service utilization. Their findings were compared with a group of people without disability identified through a population-based survey and disability records.

### Study area

One district each with poor social indicators from Andhra Pradesh (Medak district) and Karnataka (Bidar district) were included in the study. One administrative division of comparable population was randomly identified in each district (Sangareddy - Medak; Bidar taluka - Bidar).

### Sample size

The sample size was estimated using a power of 90%, significance level of 0.05, 95% confidence intervals and a difference of 25% in health care access among people with and without a disability. The estimated sample size was 1053 people with disability and an equivalent number without a disability (comparison group).

### Study methodology

The study used a two stage process to identify people with disability and age and sex-matched people without disability. In the first stage, key informants (KIs) were recruited from the study area and trained to identify people with disability. The KIs were trained using a specially designed and pretested flip book with pictorial depictions of the different impairments on identification of persons with disability, based on visible impairments/abnormalities and a brief history. KI were also oriented to the Persons with Disabilities Act (PWD Act) India, 1995 and on the disability certificates issued by the government agencies. The PWD Act includes visual impairment, hearing impairment, locomotor impairment/orthopedically handicapped, mental illness/handicap, including persons with multiple disabilities/impairments. This was important as the KI had to list persons with disabilities in their village based on the comparison with the flip book and the availability of the disability certificate. In Andhra Pradesh, the government has established a database to capture information on all persons with disability. This is called SADAREM (Software for Assessment of Disabled for Access Rehabilitation and Employment). All persons with disability who are issued a disability certificate are listed in the SADAREM database. In Karnataka information on persons with disability is available with the department of women and child development at the district headquarters.

The training was conducted in a village within the study district. All KI were transported to the training site. The duration of training was one day. Approximately 20 KIs were trained per selected block (approximately 1.5-2 persons per selected village) and their participation was voluntary, without material reward throughout the process. Each KI covered a population of between 2000–3000 over a period of 4 to 6 weeks, going house to house. At the end of 6 weeks the KI provided the list of people with disability to trained field investigators. The field investigators visited each of the persons listed by the KI. They reconfirmed the findings of the KI and simultaneously identified age and sex matched people without disability in the neighbourhood. All the identified individuals were then administered a questionnaire schedule to elicit responses regarding health care issues and employment status, in addition to recording basic demographic data. The questionnaires were translated into the local languages (Telugu and Kannada) and were pretested before use. The disability status was also ascertained from the disability certificates and disability pension records available with the people with disability. Wherever necessary, the help of the local disabled people’s organization (DPO) was solicited. In households where the person with disability could not respond due to disability, an adult responsible member of the household was asked to respond to the questionnaire (proxy respondent).

All field investigators and KI’s were people with disabilities.

In the second stage, a team of a medically trained physician and a therapist visited all listed individuals (people with and without disability) at home to confirm the diagnosis and examined them in detail for their underlying impairment and for re-ascertaining the information collected by the field investigators.

### Disabilities included in the study

Since the study used KI for the initial listing of people with disabilities, it was possible to include only those impairments which were visible to the external eye or could be picked up through a short history. The following impairments were included in the study:

- Physical impairments: Club foot, cleft lip, cleft palate, cerebral palsy, Down’s syndrome, microcephaly, phocomelia, amputated limb, burns, muscular dystrophy/atrophy, leprosy, elephantiasis, post-polio residual paralysis, congenital limb deficiencies, rickets and spinal cord injuries
- Visual Impairment: Bilateral severe visual impairment or blindness
- Hearing Impairment: Bilateral severe/profound hearing impairment
- Intellectual impairment

People with disability were defined as those who suffered from one or more of the impairments as listed as these impairments are responsible for disability due to activity limitation and effect on social participation.

All the key informants were persons with disability. Since the purpose of the study was to compare the experiences of persons with and without a disability and not to measure prevalence of disability, none of the investigators or key informants were included in the study sample to eliminate any type of measurement bias.

### Ethics

The ethical approval for the study was obtained from the Institutional Ethical Committee at Indian Institute of Public Health-Hyderabad.

### Consent

Written informed consent was obtained from all the study participants.

### Statistical analysis

The data base was developed in MS ACCESS and STATA 12.0 was used for data analysis. The chi-square test was used for associations and logistic regression was used to determine the odds for associated variables.

The age groups were categorized to reflect experience of young adults (18–29 years), middle age productive group (30–49 years), older less productive age group (50–64 years) and elderly less/non-productive age group (65+ years).

### Quality assurance

Steps were taken to assure the quality of data collected. The flip-book was pretested with the general population before being used in the study. A pilot study was conducted in two clusters which were not part of the main study. During the training, KI were made to compare their findings with the trainers and those who needed retraining were provided the same. During the entire duration of the study, the data collected by KI was verified by a team of trained senior health personnel well-versed with disability. All data was cross-checked in the field setting before being transmitted to the data entry station. A random check of the filled forms was undertaken at the data entry station by a senior investigator.

### Services

All participants were provided referral linkages to tertiary care centres for treatment wherever required. Transportation was organized for the people with disabilities to reach the tertiary centres. Treatment provided included surgery and provision of assistive devices.

## Results

A total of 57 KIs were trained in the study. They covered a total of 29 villages (13 in Sangareddy and 16 in Bidar taluk) with a total population of 100,418. A total of 839 people with disabilities and 1153 age and sex matched people without disability, aged 18 years or more were identified for the study. The mean age, sex mix and age distribution were similar in both groups (people with and without a disability) (Table 1).

**Table 1:** **Comparison of demographic characteristics of people with and without disability**

Parameter	People with disability		People without disability	
	N	%	N	%
Total (1992)	839	42.1	1153	57.9
Male (1295)	545	65.5	750	65.1
Female (697)	294	35.0	403	34.9
	Pearson chi^2^ = 0.0017; P =0.967; Not significant			
Mean Age (in years)	39.2	SD: 15.4	38.2	14.5
Age Category				
18-29 yrs	272	32.4	377	32.7
30 – 49 yrs	345	41.1	498	43.2
50-64 yrs	140	16.7	199	17.3
> = 65 yrs	82	9.8	79	6.8
	Pearson chi^2^(3) =5.7270 P =0.126; Not significant			

Among the people with disability, 88.4% (742) had a physical impairment, 4.8% (40) had severe hearing impairment, 4.5% (38) had severe visual impairment/ blindness and 2.3% (19) had intellectual impairment.

Among the persons with disability, 9.5% (80) had multiple impairments. Half of them were due to hearing impairment and speech/communication difficulties.

There were 63 (7.5%) persons where a proxy respondent from the household was required to provide answers to the questionnaire regarding barriers. Among them, 63.4% (40) were suffering from hearing impairment with or without speech or communication disorders, 30.2% (19) with intellectual impairment and 6.3% (4) were suffering from cerebral palsy (included as physical impairment).

### Employment status and concerns

There was a significant difference in the employment status of people with disability compared to people without a disability (Table 2). The difference in the two groups with respect to hours of work required, were also significant. Sex differences were observed in relation to current employment. Employment rate amongst men with disability was 61.7% compared to 47.3% among women with disability and these differences were statistically significant (Pearson chi^2^(1) -16.0610; p <0.001).

**Table 2:** **Comparison of Employment Related Issues between people with and without a disability**

Parameter	People with disability (n-839)		People without disability (n-1153)	
	N	%	N	%
Total (1992)	839	42.1	1153	57.9
Currently Not Employed	364	43.4	194	16.8
	Pearson chi^2^(1) =169.8755 P < =0.001			
Population responding to concerns regarding employment (denominator)	475		959	
Face problems with work allocated	259	54.5	433	45.1
	Pearson chi^2^(1) =11.17; p <0.001			
Face problem with hours required at work	236	49.7	353	36.8
	Pearson chi^2^(1) =21.74; p <0.001			
Need special equipment at work	175	36.8	194	20.2
	Pearson chi^2^(1) =45.84; P <0.001			
Need assistance or close supervision at work	233	49.0	339	35.3
	Pearson chi^2^(1) =24.86; P =0.001			
Find it difficult to get job they want	230	48.4	332	34.6
	Pearson chi^2^(1) =25.37; P <0.001			
Health benefits/insurance provided by employer	239	50.3	489	51.0
	Pearson chi^2^(1) =0.06; P =0.8			
Permission given by employer to go to hospital as and when need arises	249	52.4	521	54.3
	Pearson chi^2^(1) =0.46; P =0.49			

People with disability were disadvantaged significantly in relation to other aspects of employment like need for customized equipment, need for constant supervision or assistance during working hours or in getting the type of job that they would prefer (Table 2).

### Comparison of health parameters

People with disability (18.4%) needed to visit a hospital significantly more often in the preceding year compared to people without a disability (8.8%) (Pearson chi^2^ (1) =40.0562; P < =0.001) (Table 3). Significant differences were also observed with respect to hospitalization experience in their lifetime as well as current medication. All these aspects of health care access demonstrated that people with disability had a significantly higher need for health care compared to people without a disability. There were no differences in these parameters by sex of the person with disability.

**Table 3:** **Comparison of self-reported health parameters between people with and without disability**

Parameter	People with disability		People without disability	
	N	%	N	%
Total (1992)	839	42.1	1153	57.9
Needed to visit hospital in past year	154	18.4	101	8.8
	Pearson chi^2^(1) =40.0562; P < =0.001			
Ever hospitalized	149	17.8	58	5.0
	Pearson chi^2^(1) =84.4992; P <0.001			
Currently on medication	79	9.4	59	5.1
	Pearson chi^2^(1) =13.9189; P <0.001			
Suffers from diabetes	105	12.5	8	0.7
	Pearson chi^2^(1) =126.8201; P < =0.001			
Suffers from hypertension	10	1.3	10	1.0
	Pearson chi^2^(1) =1.7310 P =0.421			
Suffers from convulsions	105	12.5	8	0.7
	Pearson chi^2^(1) =126.8201; P < 0.001			
Suffers from depression	174	20.7	28	2.4
	Pearson chi^2^(1) =178.6808; P < 0.001			

The study observed that people with disability are more prone to prolonged illness than those without disability. Chronic disease indicators were significantly worse off in people with disabilities compared to those without disabilities (Table 3). A significantly higher proportion of people with disabilities stated that they suffered from diabetes, generalized convulsions and depression compared to people without disabilities. Prevalence of self-reported hypertension was similar in both groups.

Sex differences were observed in the proportion of people with disability suffering from diabetes and depression (where men were affected significantly more). The prevalence of diabetes was 14. 3% in men compared to 9.18% in women (Pearson chi^2^(1)-4.5873; p =0.032), and prevalence of depression in men was 24.2% compared to 14.3% in women (Pearson chi^2^(1)-11.4663; p = 0.001).

Adjusted Odds Ratios were computed and it was observed that people with disabilities had a 5.1 times higher risk of being hospitalized at any time compared to people without a disability (Table 4). People with disabilities had 4.6 times higher risk of suffering from diabetes and 5.8 times higher risk of suffering from depression compared to people without a disability.

**Table 4:** **Comparison of health parameters between people with and without disability (Adjusted Odds Ratios)**

Parameters	Adjusted OR*	95% CI
**Needed to visit hospital in past year**		
People without disability	1.0	
People With Disability	1.58	0.99-2.48; p = 0.05
**Ever hospitalized**		
People without disability	1.0	
People With Disability	5.1	2.63-9.9; p <0.001
**Currently on medication**		
People without disability	1.0	
People With Disability	0.95	0.58 – 1.59; p = 0.86
**Suffers from diabetes**		
People without disability	1.0	
People With Disability	4.61	1.66 – 12.81; p = 0.001
**Suffers from depression**		
People without disability	1.0	
People With Disability	5.85	3.43 – 9.98; p <0.001

*Adjusted for Age, Sex and other variables presented in table.

### Barriers to accessing health services and perceptions on quality of care

People with disability faced significantly more barriers to accessing health services compared to people without a disability (Table 5). Barriers highlighted were ignorance regarding availability of services, costs of services and transportation. Access to care was assessed by knowing where to go for treatment and cost of transportation. The differences between people with and without disabilities in relation to not knowing where to go for treatment and cost of transportation were statistically significant (Table 5).

**Table 5:** **Barriers to accessing health care**

Parameter	People with disability		People without disability	
	N	%	N	%
Total (1992)	839	42.1	1153	57.9
Faced difficulty as did not know where to go for treatment	112	13.3	23	2.0
	Pearson chi^2^(1) =99.0981; P < 0.001			
Cost of Transportation perceived as a barrier	112	13.3	25	2.2
	Pearson chi^2^(1) =94.7927; P < 0.001			
Faced difficulty in being examined because of equipment at hospital	111	13.2	24	2.1
	Pearson chi^2^(1) =95.5363; P < 0.001			
Faced difficulty because of hospital staff behavior	106	12.6	26	2.2
	Pearson chi^2^(1) =84.5499 P <0.001			
Faced difficulty because building/ facilities were not user friendly	107	12.7	27	2.3
	Pearson chi^2^(1) =83.9000; P < 0.001			

Quality of care was assessed considering whether people were comfortable with the equipment for examination, staff behavior and the physical building infrastructure. On all these quality parameters, people with disabilities were significantly disadvantaged (Table 5). Both access and quality of care were also analyzed among people with disabilities by their sex. It was observed that the differences between men and women with disabilities were not significant, demonstrating that all people with disability suffered badly when it came to accessing health care or being provided a quality health service.

## Discussion

Access to quality and timely health care is critical for all populations. For people with disabilities who may have complex health conditions it is even more important. Understanding the health needs of people with disabilities, and barriers to accessing health services is of seminal importance if governments are to be responsive to needs of people with disabilities.

The study has highlighted challenges faced by people with disability in accessing employment opportunities, accessing health care and suffering from chronic non-communicable diseases. The study is the first effort in India to bring these challenges to attention. Health status and quality of life of people with disability are of immense public health concern. It is abundantly clear that Millennium Development Goals cannot be achieved unless people with disability receive adequate attention [30]. Many countries are now working towards ensuring universal health coverage for their populations. Universal health care is a value-addition if it reduces catastrophic health expenditure and ensures equity and access to all segments of the population. People with disabilities require the same range of health services for the diagnosis and treatment of disease, or the promotion of health as people without disabilities [31]. In the absence of equal access to health care, people with disabilities are at a serious risk of delayed diagnosis and secondary co-morbidities [32].

The observations from the present study have implications on the provision of health services for people with disability. A major finding of the study was that people with disability reported visiting a health care facility significantly more often than people without a disability. Increased use of health care services by people with disabilities has also been reported from some countries earlier [11, 16–19, 24, 33, 34]. If the unmet needs of people with disability have to be met, innovative strategies will need to be devised to reach out to people with disabilities, especially women who despite having an increased need may not be able to access services. Our study points to the lack of understanding of employers regarding health needs of people with disabilities as is evidenced by perceptions of the people with disabilities. People with disabilities also expressed concerns regarding the behaviour of the staff at the health centres and the infrastructure at health facilities. This reveals a huge gap in the expectations of people with disabilities and the actual provision of services. Efforts will need to be made to bridge this gap.

People with disabilities had very high odds of suffering from chronic diseases like diabetes and depression. Very few studies have reported on the link between disability and chronic disease earlier [24, 35]. Health promotion programs and provision of affordable drugs for people with disabilities will need to be tailored to the needs of this population and by using communication channels that are appropriate and acceptable to people with disabilities.

Our study also highlights an interesting dichotomy as people with disability report using more health services, especially in-patient care while also stating that they do not receive care when they need it. Similar findings have been reported in a study from Korea [15]. This emphasizes that people with disabilities have significant unmet need for health services even though their utilization of hospital services may be higher.

We also observed that people with disabilities encountered a range of barriers in accessing health care facilities including lack of information and physical barriers, inadequate personal assistance, affordability, limitations of resources and inaccessible infrastructure and non-friendly environments. Irrespective of sex, if people had a disability, they faced barriers in accessing health services. If universal access to health care is to be ensured and if access to equitable health as enshrined in the United Nations declaration is to be effectively provided, people with disabilities will need to be empowered to unshackle the barriers to accessing health care. Creating an enabling environment for this to happen is a critical input that is needed in India, at this point in time. India ratified the UNCRPD in 2008 and has been providing a legal framework for implementation of services for people with disabilities through the adoption of the Persons with Disability Act, 1995 [36]. Unfortunately the level of awareness regarding the provisions of different services under the Act is low even among health care professionals working with people with disability in India [37]. Therefore concerted action should start with changing the knowledge and perceptions of the health care professionals themselves, if an enabling empowering environment is to be fostered in the near future.

### Limitations of the study

The present study is limited to two districts of two neighboring southern states of India and hence the findings cannot be generalized to the entire country. The labeling of a person as having a disability was based on available records and the identification of the key informants supported by a medically trained team. If resources were available, all those included in the study could have undergone a complete medical examination and this would have improved the diagnostic accuracy. In a small proportion of people with disability, a proxy respondent provided the answers as the person with disability was unable to respond on his/her own. There may be a difference in perception of a person with disability compared to the proxy respondent.

## Conclusions

This study provides evidence on the various challenges that people with disability face in accessing health-care and employment opportunities. It also highlights the increased risk of chronic non-communicable diseases that the people with disabilities face, as against people without disability. These findings have major public health implications and health planners and administrators need to keep in mind, people with disabilities, when they devise and implement strategies for better health care services. This holds good for ensuring, minimizing barriers for accessing employment opportunities too. It is also important that in all decisions concerning them, people with disability should be consulted as the essential stake holders before any intervention is finalized.
